# Invertebrate biodiversity continues to decline in cropland

**DOI:** 10.1098/rspb.2023.0897

**Published:** 2023-06-14

**Authors:** Francesca Mancini, Rob Cooke, Ben A. Woodcock, Arran Greenop, Andrew C. Johnson, Nick J. B. Isaac

**Affiliations:** UK Centre for Ecology and Hydrology, Wallingford, OX10 8BB, UK

**Keywords:** agroecosystems, agricultural policy, *Carabidae*, *Syrphidae*, *Apoidea*, *Arachnida*

## Abstract

Modern agriculture has drastically changed global landscapes and introduced pressures on wildlife populations. Policy and management of agricultural systems has changed over the last 30 years, a period characterized not only by intensive agricultural practices but also by an increasing push towards sustainability. It is crucial that we understand the long-term consequences of agriculture on beneficial invertebrates and assess if policy and management approaches recently introduced are supporting their recovery. In this study, we use large citizen science datasets to derive trends in invertebrate occupancy in Great Britain between 1990 and 2019. We compare these trends between regions of no- (0%), low- (greater than 0–50%) and high-cropland (greater than 50%) cover, which includes arable and horticultural crops. Although we detect general declines, invertebrate groups are declining most strongly in high-cropland cover regions. This suggests that even in the light of improved policy and management over the last 30 years, the way we are managing cropland is failing to conserve and restore invertebrate communities. New policy-based drivers and incentives are required to support the resilience and sustainability of agricultural ecosystems. Post-Brexit changes in UK agricultural policy and reforms under the Environment Act offer opportunities to improve agricultural landscapes for the benefit of biodiversity and society.

## 1. Introduction

Human activities have impacted the natural world in myriad ways [1], with climate and land use changes representing leading drivers of the biodiversity crisis [2]. Agricultural expansion is the most widespread form of land use change, with over one third of the terrestrial land surface being used for crops or livestock farming [2]. Agriculture is also a significant source of pollution, particularly through the use of pesticides, an additional major direct driver of biodiversity loss [2]. Over 40 000 species listed in the IUCN Red List of Threatened Species are threatened by agriculture [3], and arable farming (annual and perennial non-timber crops) accounts for risks posed to over 80% of these species [3]. The loss of biodiversity, especially organisms that provide key ecosystem services, such as pollination, pest control and decomposition, may have a significant impact on our ability to sustainably produce food to feed a growing human population [4]. Invertebrates are particularly susceptible to some farming practices, such as pesticide use and ploughing [5,6], and to the landscape-scale consequences of intensive agriculture, such as landscape simplification and the loss of wild plants and natural habitat [7]. Thus, quantifying the risks posed by agriculture on invertebrate diversity is a key question in conservation biology and crucial for targeting mitigation measures to support the long-term sustainability of agricultural systems.

Since the 1940s, agriculture management has undergone major changes driven by technological innovations and government policies—the Green Revolution. After the Second World War, agricultural policy in Europe focused on increasing the production of key commodities, which drove an expansion in the area of arable land, especially for cereal crops such as wheat and barley [8], along with an increase in the intensity of its management [8–11]. This was achieved through chemical inputs, such as pesticides and fertilizers, selective breeding of crops and large-scale mechanization, especially in more developed countries [12]. Agricultural landscapes changed significantly throughout the 1960s, 1970s and 1980s due to an increase in the size and specialization of arable farms as well as the loss of non-crop features, such as hedges and ditches [9]. Increasing the short-term productivity of agricultural systems came at the cost of their long-term resilience as these ‘simplified’ landscapes support a smaller diversity of organisms [13–18]. Many species were lost from traditional farming systems due to the loss of non-crop habitat (such as grassland and field boundaries), which provide crucial resources such as food, nesting habitat and overwintering sites. Other aspects of crop management typical of intensive farming, such as the switch from spring to winter sowing for cereals, the intensive use of agrochemicals (particularly pesticides) and cultivation methods, such as ploughing, have also been associated with a reduction in farmland biodiversity [9,19].

Since the 1990s, there has been an ongoing attempt to mitigate the impacts of intensive agriculture to help support biodiversity. In Europe, large changes in management practices have occurred since the 1990s, particularly after the 1992 reform of the Common Agricultural Policy, which required member states to develop agri-environment schemes [9]. These programmes were successful in increasing landscape heterogeneity [11], resulting in, for example, reduced rates of hedge removal in Great Britain [20], with mixed levels of success in restoring biodiversity [21,22]. In the same time period, improvements were made in the regulation and use of agrochemicals. Modern pesticides are less persistent and more efficient, so that toxicity to non-target organisms is reduced [23] and smaller amounts of active ingredient are required [8]. This has led to a reduction in the weight of active chemical used compared to the 1990s; however, the number and extent of applications has increased [8]. Therefore, the use of pesticides remains a major concern for biodiversity [5].

Given these changes in cropland farming policy and practices (cropland here referring to arable and horticultural land), we might expect that biodiversity trends over the relatively recent past (1990s onwards) may represent a relatively minor dip, plateau or even recovery, when compared to the lows associated with the post-Second World War world. However, biodiversity does not respond immediately to environmental change and lags may occur [24]. This may result in significant declines observed now that might just be an ‘extinction debt’ from dramatic changes to our landscapes that happened in the past [25]. Unfortunately, both land use and biodiversity data on the immediate post-Second World War period associated with the most intensive phase of agricultural change are often lacking. It is likely that this period represented the point where many of the more sensitive species were lost in response to landscape, policy and management changes [26]. The loss of historically ‘sensitive’ species that were once associated with agriculture could have effectively filtered communities to leave robust (if denuded) biodiversity assemblages [27,28]. For example, Redhead *et al.* [29] suggest that pollinator webs associated with arable cropping systems may be robust due to the prevalence of generalist, rather than specialist species. This may mean that the current (e.g. post-1990s) impacts of agriculture on biodiversity may be less than expected as many species currently associated with agricultural land are robust generalists, able to persist at least over recent years. There is a pressing need to understand how the current extent of agriculture impacts upon species playing a role in the provision of key ecosystem services. A fundamental question is whether species’ growth rates within communities respond to agriculture intensity or whether they are currently relatively stable across broad species complexes.

To answer these questions, we used biological records data from 1990 to 2019 to assess trends in 1535 arthropod species in response to cropland cover across Great Britain. We divide the landscape into regions of high- (greater than 50%), low- (0–50%) and no-cropland (0%) cover, as a proxy for agriculture intensity. We then compare species trends across these regions for bees, hoverflies, ground beetles, ladybirds, spiders and plant bugs. Given the relatively stable extent of cropland cover in Great Britain across 1990–2019 [30], differences in the trends of invertebrate taxa are unlikely to be associated with land use change, but instead with differences in land use intensity and historical extinction debts. The aim of this retrospective analysis was to see whether (i) terrestrial invertebrates are in decline across all British landscapes, such as might be expected from climate change or other global pressures; (ii) declines in terrestrial invertebrates are most pronounced within areas of high-cropland cover suggesting that pressures resulting from modern intensive agriculture still pose a threat to native invertebrate biodiversity and (iii) changes are consistent between different invertebrate groups, in particular those that may be considered beneficial (pollinators or natural pest control agents) or deleterious (e.g. pests). Importantly, in this study, we do not attempt to evaluate the ‘impact’ of changes in agricultural policy and practice on invertebrate populations. Without a suitable counterfactual (what would have happened if policy and practices had not changed), and without data before/after the intervention, it is not possible to determine the impact of those changes. Instead, we describe how trends in invertebrate biodiversity differ across a gradient of cropland cover.

## 2. Methods

### (a) Species data

The long history of biodiversity monitoring through citizen science in the United Kingdom has produced large datasets recording the distribution of thousands of species across the country spanning up to five decades. These data are an invaluable resource to monitor the status of biodiversity in UK agricultural systems. We collated species occurrence data for six taxonomic groups that provide key services in cropland and for which sufficient data were available. These are as follows: (i) bees (*Apoidea*), which are central place foragers and one of the most important pollinators of crops and wildflowers in the UK, currently estimated to be worth approximately £0.5 billion in the UK [31]; (ii) hoverflies (*Syrphidae*), which also contribute to crop and wild plant pollination, but also have larvae supporting pest control as well as organic matter breakdown; (iii) ladybirds (*Coccinellidae*), which represent a semi-specialist group of predators, feeding on the eggs, early instar nymphs and larvae of many insects, with importance in the control of aphids and other ‘pests’; (iv) spiders which include obligate predators of pest control value using a range of hunting strategies and potentially being capable of dispersing large distances by ballooning; (v) carabids (ground beetles; *Carabidae*), which include many generalist species of predators important for natural pest control of both insects and the seeds of arable weeds [32,33]. We also include (vi) plant bugs (around 400 species of terrestrial *Heteroptera*, including the families *Miridae*, *Lygaeidae*, *Aradidae*, *Anthocoridae*, *Berytidae*, *Nabidae*, *Reduviidae*, *Ceratocombidae*, *Cimicidae*, *Microphysidae*, *Piesmatidae* and *Tingidae*) as an example of an ecosystem disservice (herbivory of crops).

### (b) Occupancy data sources and processing

The data used in this study come from different national recording schemes and societies: the Bees, Wasps, and Ants Recording Society, the Hoverfly Recording Scheme, the UK Ladybird Survey, the Ground Beetle Recording Scheme, the Terrestrial Heteroptera Recording Scheme (plant bugs and allied species) and the Spider Recording Scheme. These volunteer recording societies operate through a membership model, where members volunteer their time to record wildlife wherever and whenever they like. Many of these schemes have been active for decades, have well-established quality assurance procedures and have produced large datasets with good national coverage [34]. The records are presence-only data containing information on what species was observed, when and where it was observed and all records are verified by taxon experts to confirm the species identification. In order to make these data suitable for analysis, we cleaned and manipulated the data following the workflow outlined in [35]. Specifically: we standardized the records to match a minimum requirement of spatial (1 km) and temporal (day) precision; we excluded records collected before 1990 and after 2019 as we were limited by the availability of land cover data and because of the higher density of biological records from 1990 onwards; we standardized the taxonomy for each taxon group by excluding all records made to a taxonomic level higher than species, although in some cases species were modelled as part of a species aggregate (due to changes in taxonomy during the period of interest). This process of data cleaning and standardization resulted in six taxonomic datasets covering 1938 species: 232 species of bee, 312 carabids, 276 hoverflies, 46 ladybirds, 367 plant bugs and 705 spiders.

We organized the cleaned data into detection histories using the function *formatOccData* from the R package *sparta* [36]. For each visit (unique combination of 1 km site and date), the detection history shows a species as either detected (1) or not detected (0). Because the data are presence-only, we used records of other species within the same taxonomic group to infer non-detections of the focal species (the target group approach [35,37]). For example, if bee species A, C and D were detected during a visit we assumed that species B was not detected. We also calculated the number of species recorded during each visit (the list length), which is a proxy for sampling effort [38].

We excluded species that were expected to produce imprecise occupancy estimates, based on data-derived thresholds described in Pocock *et al.* [39]. This step excluded species with too few records to produce a reliable trend. We also excluded all records for the Harlequin ladybird (*Harmonia axyridis*) as the trends for this alien species are driven by its rapid invasion rather than agricultural management [40]. After filtering according to these rules-of-thumb, we generated model outputs for 224 species of bees, 221 carabids, 250 hoverflies, 41 ladybirds, 264 plant bugs and 535 spiders.

### (c) Cropland regions

**Figure 1.**
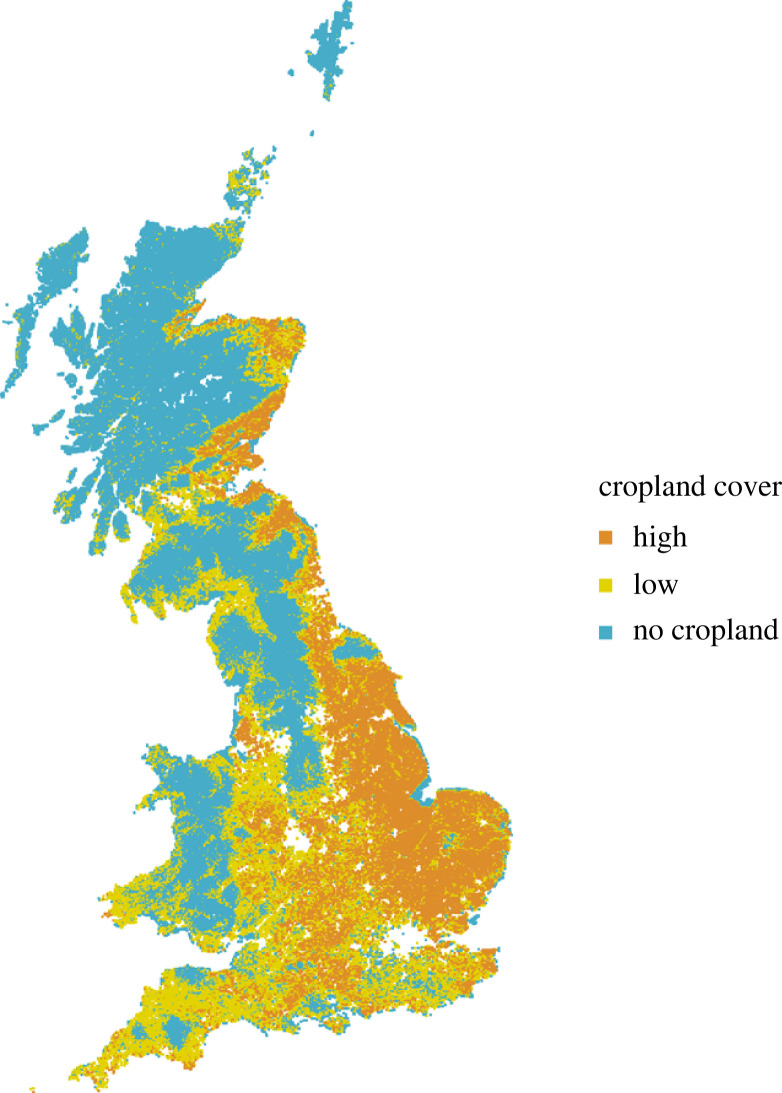
Map of cropland cover regions. One kilometre cells are classified as high if greater than 50% of the grid cell area was covered by cropland (orange; 33 149 grid cells), as low if between greater than 0% and 50% of the cell was covered by cropland (yellow; 37 885 grid cells) and as no-cropland if 0% of the cell was covered by cropland (blue; 85 553 grid cells). White represents grid cells that were excluded because a greater than 10% change in land cover was detected between 1990 and 2015 or because their land class cover class was not comparable with cropland (built-up areas, wetland and other).

We sought to classify each 1 km grid cell within Great Britain into categories of cropland cover. We used the land cover map (LCM) data for 1990 and 2015 [41,42], which include 15 land cover classes at 25 m resolution. The arable land use class in this dataset includes annual crops, perennial crops (e.g. berries and orchards) and freshly ploughed land; as this class includes both arable and horticultural crops, for simplicity, we will refer to it here as cropland. We first calculated the percentage cover for each land cover class in the 1 km cell for both 1990 and 2015. We discarded all the sites where cropland cover has changed by more than 10%, to avoid the complexity associated with land cover changes. We also excluded all sites that had a cover of greater than 25% for the land classes wetland, built-up areas and other, as they are not directly comparable with cropland. We used an empirical cumulative distribution function (electronic supplementary material, figure S1) to visualize the distribution of cropland cover across all the 1 km sites. We then used this distribution to classify sites in regions of high- (greater than 50%), low- (between 0% and 50%) and no-cropland (0%) cover (based on the LCM 2015; figure 1). Using these categories has several advantages: (i) using 50% as a threshold makes the categories easy to interpret as minority versus majority landcover; (ii) using three broad categories maximizes the power of our statistical tests as the precision of the occupancy estimates (and, therefore, our ability to detect any differences in occupancy) depends on the number of grid cells in each region and (iii) the thresholds used (0% and 50%) produced regions with approximately equal numbers of grid cells.

### (d) Occupancy models

For each species, we fitted hierarchical Bayesian occupancy-detection models [37,43] to the detection histories described above. Occupancy-detection models are made of two hierarchically coupled sub-models: one, the state sub-model, describing the ecological processes governing the true species presence or absence, and the other, the observation sub-model, describing the observation process that generated the data. This hierarchical structure allows occupancy-detection models to explicitly model imperfect detection.

We follow the occupancy-detection model from Outhwaite *et al.* [35]. The state model describes the true occupancy state, *z_it_* of site *i* on year *t* as a Bernoulli process with probability *ψ_it_*:

2.1 $$z_{it}\;∼\;\mathrm{Bernoulli}(\psi_{it}).$$

The logit of the probability of occupancy *ψ_it_* varies with year and site, and because we are interested in comparing the trends in the different regions of cropland cover, the year effect was different for the three regions of high-, low- and no-cropland cover.

2.2 $$\mathrm{logit}(\psi_{it})\;=b_{t,\mathrm{high}(i)}+b_{t,\mathrm{low}(i)}+b_{t,\mathrm{none}(i)}+u_{i},$$

where *b_t_*_,high(*i*),_ *b_t_*_,low(*i*)_ and *b_t_*_,none(*i*)_ are the year effects for year *t* in the region in which site *i* is found and *u_i_* is the site random effect on the probability of occupancy.

The observation sub-model describes the process by which the data were generated, conditional on the true occupancy state *z_it_*. This component of the model describes an observation as the product of two processes: first, a species needs to be present at the site and time of survey (*z_it_* = 1), and second, the species needs to be detected by the observer. The probability of an observer detecting a species during a visit *v*, *p_itv_*, is modelled as a function of a year effect, *a_t_*, to account for temporal variation in recording intensity, and list length (the number of species recorded). Following Van Strien *et al.* [43], we modelled detectability as a categorical function of list length, with three categories representing different data types: lists of 1, 2–3 or at least 4 species recorded. So, the observation sub-model used in here is described by the following equations:

2.3 $$y_{it}|z_{it}\;\;∼\mathrm{Bernoulli}(\;p_{itv}\times z_{it}),$$

and

2.4 $$\mathrm{logit}(\;p_{itv})\;=\;a_{t}\;+\;\beta 1\;\times \mathrm{datatype}2_{\mathrm{itv}}+\;\beta 2\;\times \;\mathrm{datatype}3_{\mathrm{itv}},$$

where the parameters *β*1 and *β*2 represent the difference in the species detection probability for a list length of 2–3 (datatype2) and 4+ (datatype3) relative to a list length of 1.

We used a random walk prior on the year effect of the state model (*b_t_*). This prior formulation allows information on species occupancy to be shared across years so that changes in occupancy are similar to that of the previous year with some variation [44], effectively allowing the year effect to change smoothly over time. Uninformative priors are set on all other parameters following [44], more details can be found in the model code.

The models were fitted using the *occDetFunc* from the R package *sparta*, which uses a Markov chain Monte Carlo (MCMC) algorithm to fit the models via JAGS. Three MCMC chains were run for 32 000 iterations with a burnin of 30 000 and a thinning rate of six.

We determined model convergence using the Gelman–Rubin statistic (Rhat), so that convergence was deemed acceptable when Rhat value was less than 1.1. The rule-of-thumb filtering retained some models with non-converged occupancy estimates. Because we were most interested in species trends, which are based on the occupancy estimates in the first and last year or the time period, we also filtered out those species for which occupancy in the first and last year showed Rhat values greater than 1.1 (248 species). We show the results from this subset of models in electronic supplementary material, figures S2 and S3. For most taxonomic groups, the magnitude of effect sizes was similar in the two subsets. The most notable difference is for the hoverflies, where the difference in annual growth rates between high- and low-cropland regions became positive when excluding species that did not converge in the first and last year. Electronic supplementary material, figure S4 shows that on average occupancy is higher in the converged subset, indicating that the convergence threshold possibly excluded many rare species. Rare species can be more vulnerable to environmental and anthropogenic pressures, which can explain why once we exclude them from the analysis the difference in trends between high-and low-cropland cover becomes positive. We decided to keep rare species into the analysis and, therefore, only show the results for the rules-of-thumb subset in the main text, for three reasons: (i) rare species are important for and often the main focus of conservation policy and action; (ii) because common and widespread species tend to be much more robust to anthropogenic and environmental pressures, only presenting the results for a subset of common species is going to provide only a partial and overly optimistic picture and (iii) once results are combined in the multi-species average, evidence from single species with uncertain estimates becomes stronger, while we propagate that uncertainty to the multi-species trend.

### (e) Inference and hypothesis testing

We first sampled 999 posterior estimates of occupancy (proportion of occupied grid cells) for all three regions of cropland cover, per species:year combination (3 regions × 1535 species × 30 years = 138 150 combinations). All subsequent analyses are based on derived parameters estimated from these data. We calculate 999 estimates for each derived parameter, thus propagating uncertainty from the species-level models up to the hypothesis tests.

The first derived parameter is the annual occupancy for each taxonomic group in each cropland cover region, which we calculated as the geometric mean across species. Next, we calculated long-term trends in occupancy of each species as the percentage annual growth rate between the first (1990) and last (2019) years of data following equation (2.5) as in Outhwaite *et al.* [35]. We then summarize the species growth rates for each taxonomic group as the geometric mean across species.

2.5 $$\mathrm{growth}\;\mathrm{rate}\;=\left(\;{\left(\frac{\;f}{s}\right)}^{1/y}-\;1\right)\;\times \;100.$$

To test whether invertebrate trends differ between regions of cropland cover, we calculated the difference (absolute effect size) between the annual growth rates in the different regions of cropland cover for each taxonomic group. Although changes in occupancy are coarse metrics of population trends, they are underpinned by changes in species' abundance [45] and are important to understand a species’s conservation status. For example, declines in area of occupancy are one of the criteria used by the International Union for Conservation of Nature for classifying a species as threatened in the Red List of Threatened Species [46].

We summarized annual occupancy, multi-species trends and absolute effect sizes across the 999 posterior estimates with the median and 95% credible interval (*HDInterval* R package [47]).

## 3. Results

A similar number of species was found in the three regions of cropland cover: 1317 in the high region, 1507 in the low region and 1485 in the no-cropland region. The high-cropland region represented 21%, low-cropland 24% and no-cropland 55% of the total area based on the proportion of 1 km grid cells assessed. Areas of high- and low-cropland cover are concentrated in the south and east, particularly in England and the east coast of Scotland, while areas of no-cropland cover are typically found in the west and north (figure 1), coinciding with cooler and wetter upland habitats, which are not suitable for crop farming. Both the habitat type and the geographical distribution of our cropland regions could affect the levels of biodiversity observed in these regions independently from their management intensity. For example, lowland regions in the UK are associated with higher species diversity in most taxonomic groups compared to upland areas [48,49]. For this reason, it was decided that in the context of the British landscape the no-cropland region did not represent a control given its tendency to be located only in areas unsuitable for crops due to differences in climate, soil, latitude and elevation [50]. Therefore, we focus our inference on the comparison between the regions of low- and high-cropland cover, which present some spatial overlap. We still present species trends for the no-cropland cover region, but we do not attempt to interpret them as a reference point for assessing the impacts of agriculture intensity.

### (a) Average occupancy and relative trends across a gradient of cropland cover

**Figure 2.**
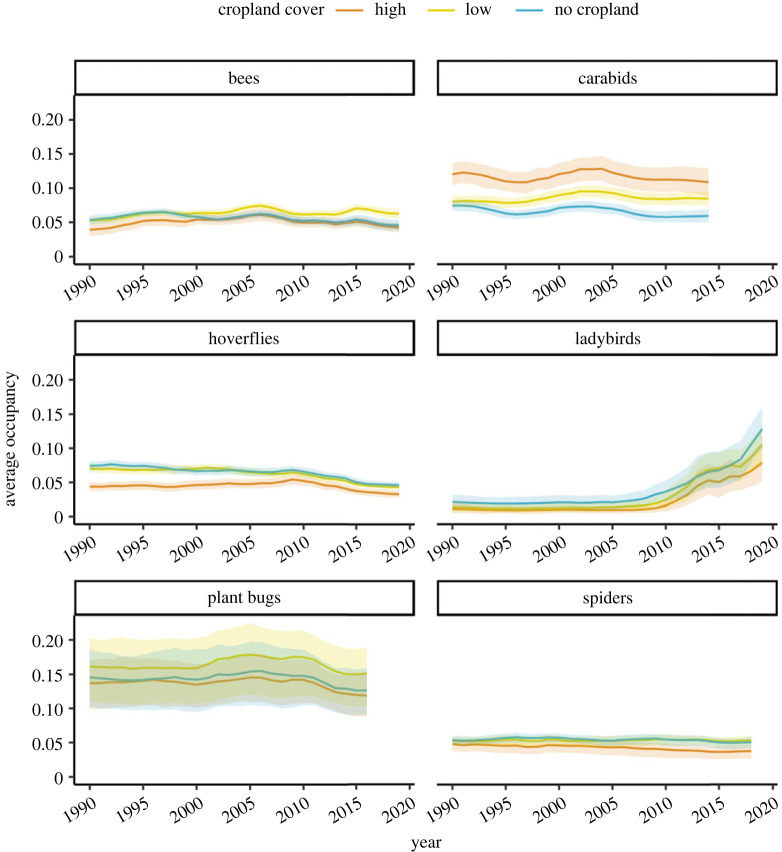
Multi-species occupancy indicator per taxonomic group. The panels show the average annual occupancy (the proportion of sites estimated to be occupied by the model) across species in the three regions of high-, low- and no-cropland cover for each taxonomic group. Lines are the mean across the posterior samples and shaded areas are 95% credible intervals.

Figure 2 shows how widespread (or rare) species within each taxonomic group are on average in the three regions of cropland cover and how average occupancy changes through time. For most taxonomic groups, levels of occupancy were higher in regions of low- and/or no-cropland cover than in areas where cropland was the dominant land cover class (figure 2). The carabids were a noticeable exception to this pattern, with average levels of occupancy in areas of high-cropland cover twice as high as those in areas without cropland (figure 2; mean_high_ = 0.12, mean_low_ = 0.08, mean_no_crop_ = 0.06). Figure 2 also shows that patterns of temporal change in occupancy differ across the six taxonomic groups.

**Figure 3.**
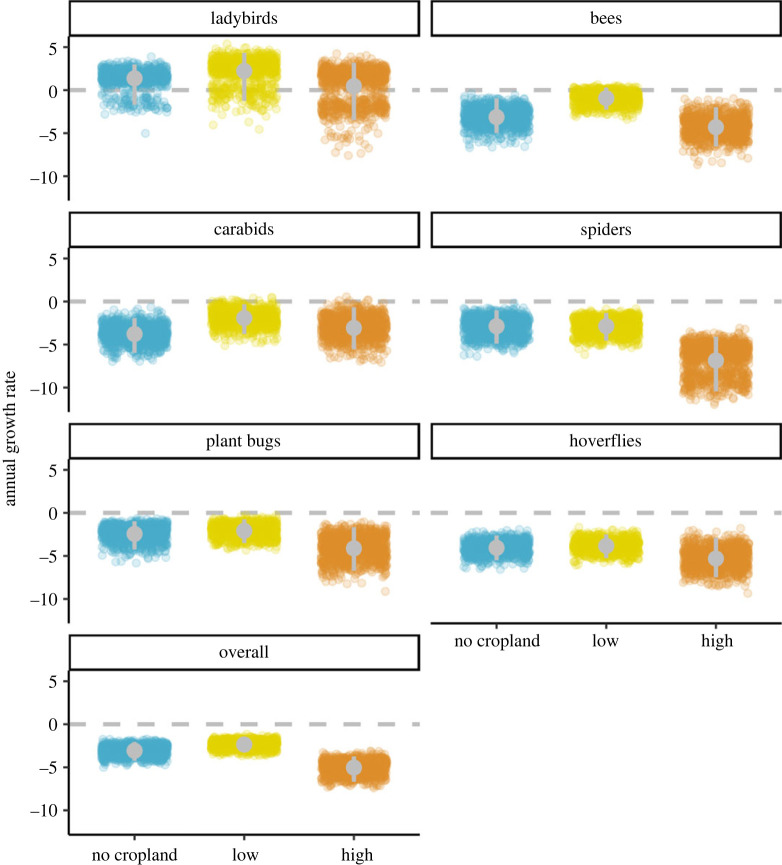
Annual growth rates from 1990 to 2019 per taxonomic group and region of cropland cover, expressed as percentage of the 1990 value. Points show 999 estimates of the geometric mean growth rate across species. Grey dots with grey vertical lines are the mean and 95% credible interval.

Growth rates since 1990 for the six taxonomic groups showed that species occupancy is generally declining both in cropland and elsewhere (figure 3). Spiders and hoverflies showed the strongest negative trends, with hoverflies declining by up to 5% since 1990 (mean_high_ = −5.3, lowCI_high_ = −7.5, uppCI_high_ = −3.0) and spiders declining by up to 7% (mean_high_ = −7.0, lowCI_high_ = −10.4, uppCI_high_ = −4.0). The trajectory of these declines differs between the taxa, with spiders showing a slow and steady decline throughout the time period, while hoverflies show a sharp decline after 2007 (figure 2). Although not as severe as in spiders and hoverflies, the growth rates of bees, carabids and plant bugs are also worrying, indicating losses up to around 4% (bees: mean_high_ = −4.3, lowCI_high_ = −6.5, uppCI_high_ = −2; plant bugs: mean_high_ = −4.1, lowCI_high_ = −6.7, uppCI_high_ = −1.6; carabids: mean_no_crop_ = −3.8, lowCI_no_crop_ = −6, uppCI_no_crop_ = −2). These taxa showed a similar pattern of decline as the hoverflies, with occupancy decreasing sharply in more recent years: 2015 for the bees, 2004 for the carabids and 2010 for the plant bugs. The exception to this general pattern of decline is the ladybirds, which increased from an average of 1.1% of sites occupied in 2010 (lowCI = 1%, uppCI = 2%), to an average of 8% (lowCI = 5%, uppCI = 12%) of sites occupied across the three regions of cropland cover in 2019 (figure 2). Despite this apparent increase in ladybirds distribution, a large part of the posterior distribution (7% across cropland regions) is still below 0, especially in areas of high-cropland cover (9%; figure 3).

### (b) Invertebrate trends in low- versus high-cropland regions

**Figure 4.**
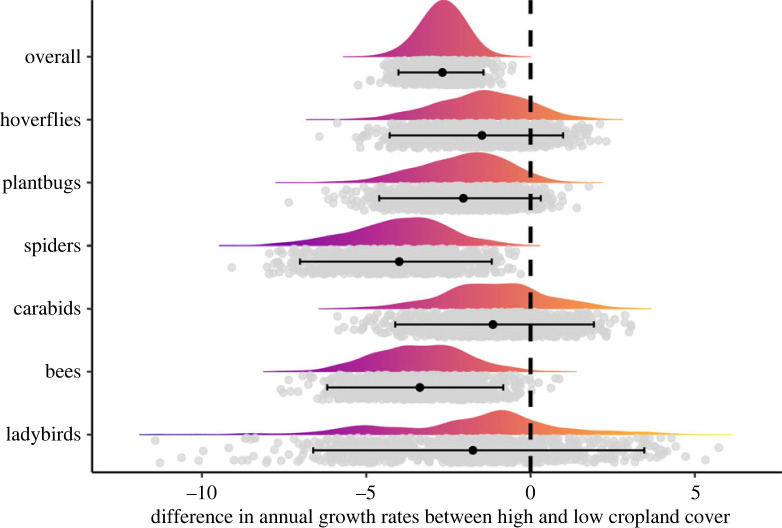
Differences in annual growth rates from 1990 to 2019 between regions of high- and low-cropland cover for each taxonomic group. Negative numbers indicate declines were more severe in areas of high cropland than in low cropland. Grey dots are the differences between growth rates in high- and low-cropland cover from the 999 posterior distribution samples. Density curves visualize the distributions of the difference values. Black dots and error bars are the mean and 95% credible intervals.

Overall species trends were more negative in areas of high-cropland cover (mean_high_ = −5, lowCI_high_ = −6.7, uppCI_high_ = −3.7) than in areas with low-cropland cover (mean_low_ = −2.3, lowCI_low_ = −3.3, uppCI_low_ = −1.5) (figures 3 and 4). This result was mainly driven by spiders, where species in areas of high-cropland cover were declining more than twice as much as in areas of low-cropland cover (mean_low_ = −2.8, lowCI_low_ = −4.5, uppCI_low_ = −1.3; mean_high_ = −7, lowCI_high_ = −10, uppCI_high_ = −4). Bees showed a similar effect size to the spiders and overall mean with a difference between growth rates in high- and low-cropland of −3.4 (lowCI = −6.2, uppCI = −0.8). We found smaller effect sizes in hoverflies and plant bugs, where the credible intervals overlapped 0, although most of the posterior distribution was negative (86% for hoverflies and 95% for plant bugs). Carabids and ladybirds showed smaller differences in growth rates between areas of high- and low-cropland cover (carabids: mean = −1.1; ladybirds: mean = −1.7), as well as greater uncertainty (carabids: lowCI = −4.1, uppCI = 1.9; ladybirds: lowCI = −6.6, uppCI = 3.5).

## 4. Discussion

### (a) Overall trends in distribution of invertebrate groups

Our analysis of biological records data for invertebrate groups providing agriculturally important ecosystem services (and disservices) in Great Britain provides evidence of general declines from 1990 to 2019 (figure 2). These results are in agreement with previous studies that have provided evidence of declines in terrestrial invertebrate diversity and abundance [51–53] and they imply that we are failing to stop, or even slow down, biodiversity loss at a national scale in Great Britain. In fact, for most of the taxonomic groups included in this study, declines appear to have accelerated over recent years (figure 2). This worsening of declines can be potentially attributed to a number of different drivers linked to, as well as beyond agricultural practices, such as changes to the types and severity of insecticides, habitat changes or loss around cropland, climate change and other weather-related events such as droughts, as well as potential interactions and synergies between drivers. Moreover, the response of insects to these potential drivers could be lagged by varying amounts [54], making driver attribution difficult. As this is a purely descriptive study, we are not able to identify which environmental and/or policy drivers may be behind this pattern or rates of recent declines.

Importantly, differences in these declining trends between the different cropland cover classes are small relative to differences between the taxa investigated (figure 2). To a large extent this reflects the unique characteristics of individual species that result in large variation in sensitivity to agriculture, such as aspects of local and landscape-scale resource exploitation [7], species mobility, habitat specialization and behavioural and physiological traits. This variability in responses to landscape characteristics among different invertebrate taxa is well documented [55,56], with meta-analyses often failing to derive general patterns across these groups [57]. This unexplained variation can be linked to a failure to capture spatial and temporal variables at the scale relevant for the organisms, as well as species-specific effects due to unique trait characteristics (e.g. specialists versus generalists) or trophic interactions [55]. While our proxy for agricultural intensity (percentage bands of cropland cover) may fail to capture the nuances of spatial land use structure and temporal patterns in management evolution, this study still provides evidence for the long-term negative effects of agriculture as an aggregate driver of biodiversity changes in invertebrate taxa. Importantly this remains an issue even after the changes in policy and management practices that occurred in the last 30 years. Although this study cannot evaluate the impact of these changes, the fact that invertebrate biodiversity is still declining in cropland suggests that current policies and practices are not providing adequate protection.

### (b) The influence of cropland on invertebrate trends

Over and above this general invertebrate decline, we have shown that this loss was greatest in areas of higher cropland cover (figure 4). As cropland cover increases within a site, refuge habitats and complementary and supplementary food resources become scarce and/or are more dispersed over a landscape characterized by an increasingly impermeable habitat matrix, dominated by intensively managed crop fields [58,59]. This can exacerbate the impacts of intensive agricultural management practices, affecting invertebrates directly or indirectly when compared to communities that have access to high-quality habitat within their dispersal distance. There is much evidence that insecticide and herbicide use is a strong driver of declines in farmland invertebrates [5,6]; however, pesticides are clearly not the only culprit, with habitat and landscape complexity but also being important drivers of invertebrate richness and abundance in agricultural systems [7]. Based on the results presented here, we are not able to determine which agricultural practices are driving these declines and we can only describe the trends observed in areas with different percentage cover of cropland.

This pattern of stronger declines in areas of high-cropland cover was evident for most taxa (figure 3), although the effect size and uncertainty varied across groups (figure 4). Overall, the strongest evidence for the negative effects of agriculture was for bees and spiders (figures 3 and 4). Differences in the way the species within each taxon use the landscape may explain some of this variability (electronic supplementary material, figure S5). Around 80% of bee and spider species are dependent on semi-natural habitat as they cannot find all the resources they need in cropland [60]. This is in contrast with other taxa, for example, hoverflies and carabids, where a higher proportion of species are not dependent on semi-natural habitat (31% and 56%, respectively [60]). Many species of ladybirds and some hoverflies are specialist aphidophagous predators and may be able to use widely occurring aphid colonies on wild plants not actively treated with pesticides even in intensively managed landscapes.

Our models include some for which the occupancy estimates did not achieve satisfactory convergence. When we excluded these, the mean difference in annual growth rates between areas of high- and low-cropland cover was closer to 0 (electronic supplementary material, figure S2). As this subset of models excludes most of the rare species (electronic supplementary material, figure S3), this result suggests that common and widespread species are less sensitive to the pressures of intensive agriculture, as already documented in previous studies [27–29]. Interestingly, although differences in annual growth rates between areas of high- and low-cropland cover were smaller for this subset of species and credible intervals overlapped 0, they were still negative for most taxonomic groups, therefore supporting the general pattern (electronic supplementary material, figure S2).

### (c) Invertebrate trends outside cropland

We intended to use the no-cropland category as a control; however, due to the confounding effect of climate and geography, we would not be making a fair comparison. This reflects historic and bio-climatic drivers of arable and horticultural expansion in Great Britain such that the no-cropland region was predominantly in upland areas (figure 1) [50]. Moreover, the distribution of our cropland regions overlaps with a north–south axis, which can further confound the results, as most no-cropland sites are in the north, while the majority of sites with low- and high-cropland cover are in the south. Much of this land is in fact agricultural, but pasture rather than arable, either intensively (typically lowland) or extensively (typically upland) managed. Biodiversity is not distributed homogeneously in space [61] and neither are drivers of environmental change [62]. When comparing species trends with those found in the no-cropland region, it wasn't possible to distinguish the effect of land cover from the effect of climate and other drivers affecting upland landscapes. For example, the upland landscapes of Scotland, which make up the majority of the no-cropland region, are affected by different anthropogenic and environmental drivers, such as intensive sheep and deer grazing, afforestation for commercial forestry, acid and nutrients atmospheric deposition and climate change [63,64]. Moreover, because the majority of no-cropland sites are in the north of the country (figure 1), populations in this region may also be at the edge of their ranges and so more sensitive to environmental changes and susceptible to extinction [65].

Figure 1 also shows that areas of high-cropland cover tend to be predominantly in the east compared to areas of low-cropland cover (electronic supplementary material, figure S6). Consequently, it is possible that these two regions differ in some environmental characteristics, such as hydrology or soil. These different environmental conditions could influence biodiversity trends for reasons that are independent from agricultural practices, but we cannot experimentally control for these factors at such large scales. Although we have not attempted to disentangle the effects of all of these factors, we note that a large number of sites within the low-cropland cover class are found at similar elevations, latitudes and longitudes to sites within the region of high-cropland cover. This makes it possible to compare species trends between these two regions and distinguish greater declines within high- rather than low-cropland cover (electronic supplementary material, figure S6). Still, we cannot attribute all of this difference in trends to cropland cover, with other factors probably playing a role.

## 5. Conclusion

Changing modern agriculture from a purely production and profit driven system to one with a greater emphasis on sustainability has been a slow process [9,21,66]. While initially European drivers of change centred on the introduction of agri-environmental policy, the efficacy of these has been debated ever since [21,66,67]. Although the regulatory framework for pesticides has improved dramatically over the last 30 years, resulting in the loss or removal of many products with unacceptable risk for non-target wildlife, this remains an imperfect system. Consequences of hard to predict chronic and synergistic effects remain an ongoing problem [68,69]. Recent shifts in policy towards zero carbon strategies of farming, while welcome [70], may also have unexpected consequences for biodiversity due to their change in emphasis. While there are incentives for farmers to adopt more sustainable practices, these results suggest that all of these policy and management changes have not yet achieved the goal of bending the curve of biodiversity loss. This is an area that remains in rapid transition, and while the evidence that we present suggests it is not working, the lag between agriculture conservation efforts and population responses makes assessing this over recent time periods difficult [71]. As the pressure for food production will only increase in the future, there is an ongoing need to review the efficacy of current policy in light of direct evidence, such as that presented here, and consider what additional measures are needed to reverse or at least arrest terrestrial invertebrate decline. The exit of the UK from the European Union has brought changes in agricultural policy, including the Environment Act, which passed into UK law in November 2021. Reforms under this new legal framework offer opportunities to introduce new policy-based drivers and incentives to support the long-term sustainability of agricultural ecosystems for the benefit of biodiversity and society.

## Data Availability

The raw species data used in the analyses were provided by multiple recording schemes (Bees, Wasps and Ants Recording Society, the Hoverfly Recording Scheme, the UK Ladybird Survey, the Spider Recording Scheme, the Ground Beetle Recording Scheme and the Terrestrial Heteroptera Recording Scheme—Plant bugs and allied species) via the Biological Records Centre (BRC), Wallingford (https://www.brc.ac.uk/). Data requests should be directed to the BRC. The Land Cover Map data are available from the Environmental Information Data Centre, https://catalogue.ceh.ac.uk/documents/bb15e200-9349-403c-bda9-b430093807c7 and https://catalogue.ceh.ac.uk/documents/1be1912a-916e-42c0-98cc-16460fac00e8. Samples from the model-derived posterior distributions are available from the Environmental Information Data Centre at https://catalogue.ceh.ac.uk/documents/28538610-0936-48b8-b167-ee6ee8fdc491. The R code for the occupancy models and statistical analyses are available online at https://github.com/FrancescaMancini/AgriTrends.
