# Correction to: ‘Invertebrate biodiversity continues to decline in cropland’ (2023), by Mancini *et al.*

**DOI:** 10.1098/rspb.2023.2650

**Published:** 2024-02-07

**Authors:** Francesca Mancini, Rob Cooke, Ben A. Woodcock, Arran Greenop, Andrew C. Johnson, Nick J. B. Isaac

*Proc. R. Soc. B* **290**, 20230897 (Published online 7 June 2023). (https://doi.org/10.1098/rspb.2023.0897)

We have found some mistakes in Mancini *et al.* [1]. These mistakes make the results presented in the paper inaccurate. Below, we describe the mistakes and how we have corrected them:

1. The trends in occupancy for the ladybirds are inaccurate. In 2017, a new field guide for UK ladybird species was published, which greatly increased the recording effort for this group. In particular, the field guide boosted recording (and hence detectability) for species of inconspicuous ladybirds, which, prior to the release of this field guide, had been too difficult to identify in the field and were, therefore, under-recorded. We now believe the strong increases in ladybird occupancy shown in figure 2 of the article are an artefact of increased detectability. In our correction, we, therefore, removed all records for inconspicuous ladybirds as well as all records after 2017 and recalculated the occupancy trends.
2. We found a mistake in the code: in the calculation of the annual growth rates (as shown in figure 3 of the article), we used the arithmetic mean instead of the geometric mean to summarize growth rates across species. We have corrected this mistake and recalculated the group growth rates using the geometric mean.

**Figure 2.**
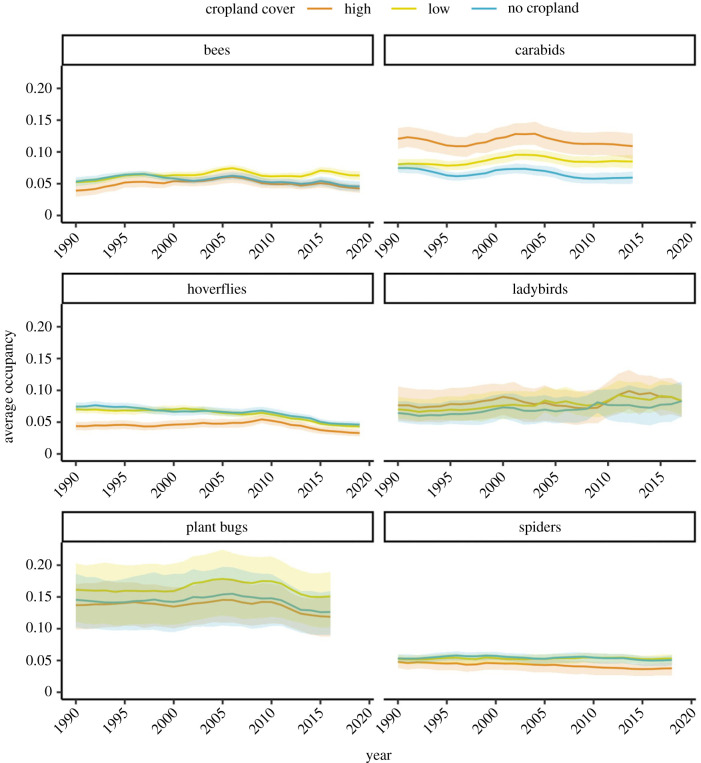
Multispecies occupancy indicator per taxonomic group. The panels show the average annual occupancy (proportion of sites estimated to be occupied by the model) across species in the three regions of high-, low- and no-cropland cover for each taxonomic group. Lines are the mean across the posterior samples and shaded areas are 95% credible intervals.

**Figure 3.**
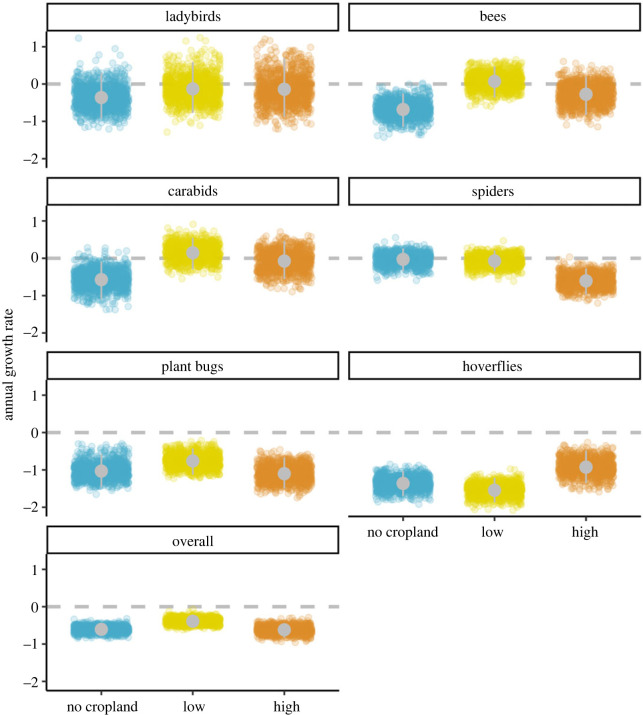
Annual growth rates from the first to the last year per taxonomic group and region of cropland cover, expressed as percentage of the first-year value. Points show 999 estimates of the geometric mean growth rate across species. Grey dots with grey vertical lines are the mean and 95% credible interval.

Correcting these mistakes has changed the numerical details of our results as described below. However, the main conclusions are not affected.

As reported in the original paper, invertebrate groups are still found to be declining across Great Britain. As previously reported, overall declines remain stronger in areas of high-cropland cover than in areas of low-cropland cover. This result, as previously, is mainly driven by the spiders, which show the strongest effect size. Our main conclusion that the last 30 years of changes to agricultural policy and practice attempting to mitigate for the negative impacts of intensive agriculture have not succeeded at reversing or stopping invertebrate declines in cropland is still valid.

However, as a consequence of the corrections, there are some changes in the nuance of the results, which are summarized below:

— Overall, annual growth rates are still negative for most groups in the high-cropland cover regions, as was previously reported, but the magnitude of declines is slightly smaller.
— Species are still generally declining more strongly in areas of high-cropland cover than in areas of low-cropland cover. This includes an overall cross-taxa effect. The revised analysis does, however, indicate that hoverflies may now be increasing in response to high-cropland cover, whereas the previous analysis suggested they were on average unaffected.
— The spiders are still the group that shows the strongest effect of cropland cover on annual growth rates, although the declines are less severe than reported in the published paper.
— Bees still show stronger declines in areas of high-cropland cover than in areas of low-cropland cover, but the 95% credible interval for the effect size overlaps zero.

Below, we present corrected text from the sections in the original paper that needed to be updated: the fourth sentence in the Abstract, the first sentence in the Results section, the sections ‘Average occupancy and relative trends across a gradient of cropland cover’ and ‘Invertebrate trends in low- versus high-cropland regions’ from the Results and the beginning of the second paragraph of the subsection ‘The influence of cropland on invertebrate trends’ from the Discussion. All changes are highlighted in bold. We also present corrected figures 2–4 from the paper and figures S2, S3 and S5 from the electronic supplementary material [2]. Finally, we present a new table (electronic supplementary material, table S1) listing the species of inconspicuous ladybirds that have been excluded from the analysis.

**Figure 4.**
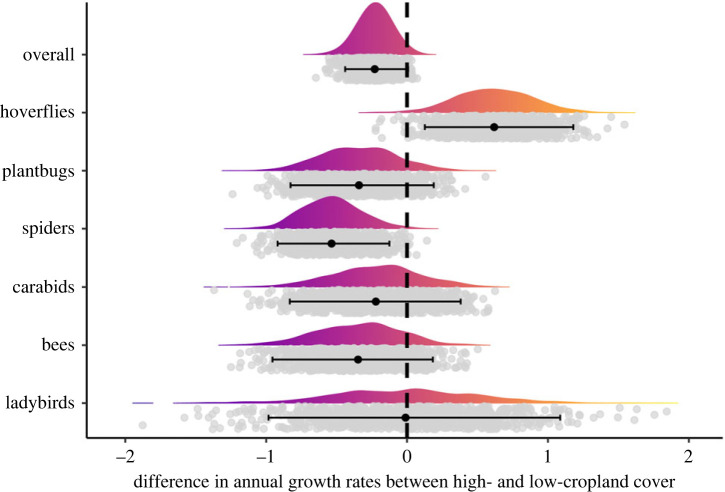
Differences in annual growth rates from the first to the last year between regions of high- and low-cropland cover for each taxonomic group. Negative numbers indicate declines were more severe in areas of high-cropland than low-cropland. Grey dots are the differences between growth rates in high- and low-cropland cover from the 999 posterior distribution samples. Density curves visualize the distributions of the difference values. Black dots and error bars are the mean and 95% credible intervals.

## Abstract

Although we detect general declines **across GB, when comparing cropland,** invertebrate groups are declining **more** strongly in high- **than low-**cropland cover regions.

## Results

A similar number of species was found in the three regions of cropland cover: **1305** in the high region, **1493** in the low region and **1472** in the no-cropland region. **As a result of excluding inconspicuous ladybird species from the analysis** **(electronic supplementary material,** **table S1), we now present the results for 27 species of ladybirds (previously 41)**.

### Average occupancy and relative trends across a gradient of cropland cover

Figure 2 shows how widespread (or rare) species within each taxonomic group are on average in the three regions of cropland cover and how average occupancy changes through time. For most taxonomic groups, levels of occupancy were higher in regions of low- and/or no-cropland cover than in areas where cropland was the dominant land cover class (figure 2). The carabids were a notable exception to this pattern, with average levels of occupancy in areas of high-cropland cover twice as high as those in areas without cropland (figure 2; mean_high_ = 0.12, mean_low_ = 0.08, mean_no_crop_ = 0.06). Figure 2 also shows that patterns of temporal change in occupancy differ across the six taxonomic groups.

Growth rates since 1990 for the six taxonomic groups showed that species occupancy is generally declining both in cropland and elsewhere (figure 3). **Hoverflies and plant bugs** showed the strongest negative trends, with hoverflies declining by up to **1.5%** since 1990 **(mean_low_ = −1.54, lowCI_low_ = −1.87, uppCI_low_ = −1.21)** and **plant bugs** declining by up to **1% (mean_high_ = −1.1, lowCI_high_ = −1.49, uppCI_high_ = −0.67).** The trajectory of declines differs between the taxa, with spiders showing a slow and steady decline throughout the time period, while hoverflies show a sharp decline after 2007 (figure 2). Although not as severe as in **plant bugs** and hoverflies, **we found declines in** the growth rates of bees, carabids, **ladybirds and spiders**, indicating losses up to around **1% (bees: mean_no_crop_ = −0.7, lowCI_high_ = −1.15, uppCI_high_ = −0.25; spiders: mean_high_ = −0.6, lowCI_high_ = −0.97, uppCI_high_ = −0.27; carabids: mean_no_crop_ = −0.57, lowCI_no_crop_ = −1.1, uppCI_no_crop_ = −0.08)**. **Bees, carabids and plant bugs** showed a similar pattern of decline as the hoverflies, with occupancy decreasing sharply in more recent years: 2015 for the bees, 2004 for the carabids and 2010 for the plant bugs. The exception to this general pattern of decline is the ladybirds, **which showed a weak negative growth rate, with the 95% CI crossing zero (mean_no_crop_ = −0.36, lowCI_no_crop_ = −0.91, uppCI_no_crop_ = 0.31)** (figure 2). Despite this, a large part of the posterior distribution (**74%** across cropland regions) is still below zero (figure 3).

### Invertebrate trends in low- versus high-cropland regions

Overall species trends were more negative in areas of high-cropland cover **(mean_high_ = −0.53, lowCI_high_ = −1.5, uppCI_high_ = 0.15)** than in areas with low-cropland cover **(mean_low_ = −0.38, lowCI_low_ = −1.71, uppCI_low_ = 0.42)** (figures 3 and 4). This result was mainly driven by spiders, where species in areas of high-cropland cover were declining more than **eight times** as much as in areas of low-cropland cover **(mean_low_ = −0.07, lowCI_low_ = −0.35, uppCI_low_ = 0.2; mean_high_ = −0.6, lowCI_high_ = −0.97, uppCI_high_ = −0.27)**. Bees and **plant bugs** showed a similar effect size to the spiders, **although the credible intervals overlapped zero (bees: mean_high-low_ = −0.34, lowCI_high-low_ = −0.95, uppCI_high-low_ = 0.18; plant bugs: mean_high-low_ = −0.34, lowCI_high-low_ = −0.82, uppCI_high-low_ = 0.18); however, most of the posterior distribution was negative (87% for bees and 89% for plant bugs**). We found smaller effect sizes in **ladybirds and carabids (carabids: mean_high-low_ = −0.22; ladybirds: mean_high-low_ = −0.01)**, as well as greater uncertainty **(carabids: lowCI_high-low_ = −0.83, uppCI_high-low_ = 0.38; ladybirds: lowCI_high-low_ = −0.98, uppCI_high-low_ = 1.08)**. **Hoverflies were the only group for which we found stronger declines in areas of low-cropland cover (mean_low_ = −1.54, lowCI_low_ = −1.87, uppCI_low_ = −1.21) than in areas of high-cropland cover (mean_high_ = −0.92, lowCI_high_ = −1.35, uppCI_high_ = −0.48).**

## Discussion

### The influence of cropland on invertebrate trends

This pattern of stronger declines in areas of high-cropland cover was evident for most taxa (figure 3), although the effect size and uncertainty varied across groups (figure 4). Overall, the strongest evidence for the negative effects of agriculture was for **spiders** (figures 3 and 4).
